# Transcriptome changes in the phenylpropanoid pathway of *Glycine max *in response to *Pseudomonas syringae *infection

**DOI:** 10.1186/1471-2229-6-26

**Published:** 2006-11-03

**Authors:** Gracia Zabala, Jijun Zou, Jigyasa Tuteja, Delkin O Gonzalez, Steven J Clough, Lila O Vodkin

**Affiliations:** 1 Department of Crop Sciences, University of Illinois, Urbana, Illinois 61801, USA; 2 USDA-ARS, Urbana, Il 61801, USA

## Abstract

**Background:**

Reports of plant molecular responses to pathogenic infections have pinpointed increases in activity of several genes of the phenylpropanoid pathway leading to the synthesis of lignin and flavonoids. The majority of those findings were derived from single gene studies and more recently from several global gene expression analyses. We undertook a global transcriptional analysis focused on the response of genes of the multiple branches of the phenylpropanoid pathway to infection by the *Pseudomonas syringae *pv. *glycinea *with or without the avirulence gene *avrB *to characterize more broadly the contribution of the multiple branches of the pathway to the resistance response in soybean. Transcript abundance in leaves was determined from analysis of soybean cDNA microarray data and hybridizations to RNA blots with specific gene probes.

**Results:**

The majority of the genes surveyed presented patterns of increased transcript accumulation. Some increased rapidly, 2 and 4 hours after inoculation, while others started to accumulate slowly by 8 – 12 hours. In contrast, transcripts of a few genes decreased in abundance 2 hours post inoculation. Most interestingly was the opposite temporal fluctuation in transcript abundance between early responsive genes in defense (*CHS *and *IFS1*) and *F3H*, the gene encoding a pivotal enzyme in the synthesis of anthocyanins, proanthocyanidins and flavonols. F3H transcripts decreased rapidly 2 hours post inoculation and increased during periods when CHS and IFS transcripts decreased. It was also determined that all but one (*CHS4*) family member genes (*CHS1*, *CHS2*, *CHS3*, *CHS5*, *CHS6 and CHS7/8*) accumulated higher transcript levels during the defense response provoked by the avirulent pathogen challenge.

**Conclusion:**

Based on the mRNA profiles, these results show the strong bias that soybean has towards increasing the synthesis of isoflavonoid phytoalexins concomitant with the down regulation of genes required for the synthesis of anthocyanins and proanthocyanins. Although proanthocyanins are known to be toxic compounds, the cells in the soybean leaves seem to be programmed to prioritize the synthesis and accumulation of isoflavonoid and pterocarpan phytoalexins during the resistance response. It was known that CHS transcripts accumulate in great abundance rapidly after inoculation of the soybean plants but our results have demonstrated that all but one (*CHS4*) member of the gene family member genes accumulated higher transcript levels during the defense response.

## Background

A common bacterial disease of soybean worldwide is the bacterial blight caused by *Pseudomonas syringae *pv. *glycinea *(*Psg*). The interactions of compatible and incompatible races of *Psg *with different soybean cultivars have been characterized previously [1]. Compatible interactions allow bacterial growth within the host and disease development, whereas incompatible interactions restrict bacterial multiplication with minimal symptom development through the sacrifice of very few cells in the immediate vicinity of the pathogen by programmed cell death. Incompatible interactions lead to a cascade of plant responses triggered by the action of a resistance gene R and the corresponding avirulent pathogen *avr *gene, which is known as the hypersensitive response (HR) [2,3].

The complex resistance response provoked in such incompatible plant-pathogen interactions have been studied and characterized at the molecular level to a large extent in the model plant *Arabidopsis thaliana *(reviewed in [4]) and to a lesser extent in agronomically important crops [5]. The inducible defense mechanisms may be local or systemic. Local defenses usually involve necrotic changes initiated by ion flux in and out of the cell, followed by the oxidative destruction of cell components by lipid hydroperoxides and reactive oxygen species, in addition to the accumulation of toxic metabolites such as phytoalexins and other phenolic compounds. Systemic defenses result in the accumulation of anti-microbial compounds in parts of the plant distant from the site of infection. Among these defenses are pathogenesis related proteins (PR), defensins, proteinase inhibitors and cell wall components such as hydroxyproline-rich glycoproteins (HRGP) and lignin and its precursors. Additionally, synthesis of salicylic acid (SA), a signal molecule that regulates systemic and local pathogen-induced defense gene activation, oxidative burst, and pathogen-induced cell death, increases [6].

**Figure 1:**
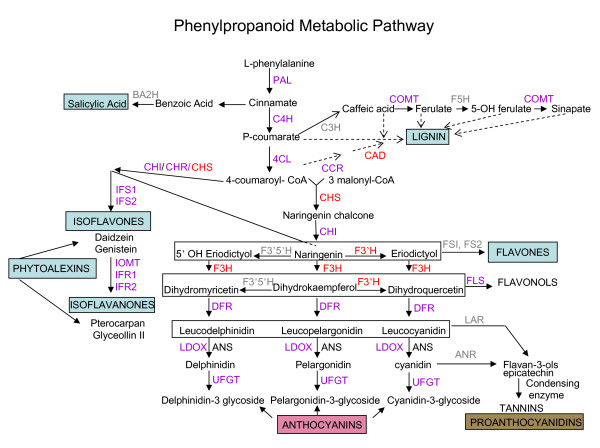
**Phenylpropanoid metabolic pathway**. Enzymes are indicated in uppercase letters. In purple are the enzymes for which cDNAs were printed in the soybean microarrays and their RNA profiles were determined in the microarray experiments. In red are the enzymes which RNA profiles were measured by microarrays and RNA blots using specific cDNA probes. In gray are enzymes for which no annotated EST exists in soybean public databases. PAL, phenylalanine ammonia-lyase; C4H, cinnamate 4-hydroxylase; 4CL, 4-coumarate: CoA ligase; CHS, chalcone synthase; CHI, chalcone isomerase; IFS, isoflavone synthase; F3'H, flavonoid 3'-hydroxylase; F3', 5'H, flavonoid 3',5'-hydroxylase; F3H, flavanone 3-hydroxylase; DFR, dihydroflavonol-4-reductase; ANS, anthocyanidin synthase also called LDOX, leucoanthocyanidin dioxygenase); UFGT, UDP-flavonoid glucosyltransferase; BA2H, benzoic acid 2-hydroxylase; C3H, p-coumarate 3 hydroxylase; COMT, caffeic O-methyltransferase; F5H, ferulic acid 5-hydroxylase; CCR, cynnamoyl CoA reductase; CAD, cynnamyl alcohol dehydrogenase.

Consequently, many secondary metabolites derived from multiple branches of the phenylpropanoid pathway, including lignins, isoflavonoid-phytoalexins, other phenolic compounds and SA are instrumental in the plant's ability to mount successful defenses to invading pathogens (Figure 1). Most studies of the phenylpropanoid pathway to date have investigated the molecular response of individual genes of the pathway. No major systematic or global analysis focused on the many genes from the multiple branches of the pathway (Figure 1) has been reported. Although general, global transcriptome changes during defense responses of various plants (*Arabidopsis*, tomato, *Medicago truncatula*, soybean) to several pathogens (*Pseudomonas spp*, *Alternaria brassicicola*, *Xanthomonas*, *Phytophthora*) have been examined [7-11], none of the analyses have thoroughly mined the data with specific emphasis on the overall response of the phenylpropanoid pathway genes. Nevertheless, those global studies have shown several groups of up regulated ESTs representing three main branches of the phenylpropanoid pathway and it appears that all plants examined respond to infection with the induction of phenylalanine ammonia-lyase (*PAL*) coumarate CoA-ligase (*C4H*) and cynamyl alcohol dehydrogenase (*CAD*) genes. Chalcone synthase (CHS) a central enzyme in the pathway is consistently induced in all plants examined with the exception of *Arabidopsis *[12]. In addition, four isoflavonoid pathway ESTs were up-regulated in *Medicago truncatula *and soybean [9,10] and four putative anthocyanin pathway ESTs in tomato [8].

CHS is the key enzyme diverting the substrate, naringenin chalcone to the flavonoid and isoflavonoid branches of the phenylpropanoid pathway that synthesizes the precursor of a large number of secondary metabolites, including proanthocyanidins, anthocyanins, flavones, flavonols and isoflavonoid-phytoalexins among others (Figure 1). Unlike *Arabidopsis *where only one chalcone synthase (*CHS*) gene exists [13] and the isoflavonoid branch of the pathway appears to be non-existent [14,15], legumes have multiple family member genes. Soybean plants have an 8-member CHS family and exhibit tissue compartmentalized expression of the isoflavonoid pathway leading to the synthesis of isoflavones in the roots and cotyledons and an inducible isoflavonoid-phytoalexins synthesis in the leaves of pathogen stressed plants [16,17].

Here we report our findings on the levels of transcript abundance of 19 phenylpropanoid pathway genes and the identification of stress-responsive *CHS *gene family member(s) in leaves of soybean (cultivar Williams 82) challenged with *Pseudomonas syringae *pv. *glycinea *with or without the *avrB *gene. Transcript abundance patterns obtained using RNA from infected leaves in hybridizations to soybean cDNA microarrays [11] were, supported and extended further by single gene RNA gel blots for five genes of key phenylpropanoid pathway enzymes (CAD, CHS, IFS, F3'H and F3H). Real time quantitative RT-PCR was also used to measure the relative transcript levels of the individual *CHS *gene family members in leaf tissues 8 hrs after inoculation.

An increase in transcript abundance was observed for most of the analyzed genes with the exception of transcripts of genes whose products function downstream of chalcone isomerase (CHI) and are required for the synthesis of flavonols, anthocyanins and proanthocyanidins (F3H, DFR, LDOX, UFGT and FLS). These decreased in abundance as early as 2 hrs post-inoculation. The more frequent transcript measurements along the 53 hrs post inoculation period in RNA gel blots detected temporal fluctuation in transcript abundance for three genes *CHS*, *IFS *and *F3H*. A significant finding was the opposite fluctuation in F3H transcript accumulation compared to that of CHS and IFS transcripts, revealing that *F3H *and other downstream genes in the anthocyanin/proanthocyanidin/flavonol pathways are underexpressed during pathogen challenge. Thus, our data suggests that flavonols, proanthocyanidins and anthocyanins are not recruited to mount the hypersensitive response in soybean leaves and that their synthesis is in competitive disadvantage with that of the isoflavonoid-phytoalexins. Interestingly, F3'H transcripts accumulate continuously 12 hrs post inoculation suggesting that this branch of the pathway is participating in the reaction cascade elicited by the defense response and possibly shifting the pathway towards the synthesis of flavones. Finally, we also found that all genes of the CHS family (*CHS1*, *CHS2*, *CHS3*, *CHS5*, *CHS6 and CHS7/8*) except *CHS4 *accumulated higher transcript levels during the hypersensitive defense response (HR) triggered by the avirulent pathogen.

## Results

### Transcript profiles of eighteen soybean phenylpropanoid pathway genes during the early response to *Pseudomonas syringae *pv *glycinea *infection

In an earlier study undertaken to analyze a global differential gene expression during the resistant (HR) versus susceptible responses in leaves of soybean plants inoculated with *Psg *with or without *avrB*, soybean cDNA microarrays [18] were used [11]. Among the 27,648 cDNAs analyzed there was a subset of cDNAs representing 19 genes of the phenylpropanoid pathway in soybean as diagramed in Figure 1. The resulting transcript profiles of this cDNA subset were examined here in further detail and compared to one another to gain an understanding of the timing in transcript accumulation changes occurring during the early hours post inoculation.

Table 1 summarizes the hybridization ratios resulting from comparisons between dual hybridizations of RNAs extracted from leaves of plants infiltrated with *Psg *carrying *avrB *(HR) and compared to those RNAs from plants infiltrated with MgCl_2 _at three time points post inoculations (2, 8 and 24 hrs) and labeled as T2HR, T8HR, and T24HR respectively. Hybridization ratios greater than 1.5 fold are written in bold type indicating an increase in transcript accumulation due to the plant's hypersensitive response to the avirulent pathogen (*Psg*) (rather than to the stress provoked by the infiltration process itself) while those in italics indicate a 1.5 fold or greater decrease (<0.67x) in the transcript abundance between the two treatments. The hybridization ratios resulting from comparing RNAs of leaves infiltrated with *Psg *lacking *avrB *(virulent strain) to RNAs from plants infiltrated with MgCl_2 _also at three time points post inoculation (2, 8, and 24 hrs) are labeled as T2VIR, T8VIR, and T24VIR (Table 1). With some exceptions, increases in transcript abundance were found in both conditions, but were generally more robust in plants undergoing the HR mediated by the avirulent pathogen than in those showing the susceptible response to the virulent pathogen strain.

For most of the 19 genes (Table 1, 1^st ^column) multiple ESTs representing a given gene or gene family were printed in the soybean microarrays. The gene description of each EST was based on GenBank assignments [18] and TIGR (The Institute for Genomic Research) databases annotations as well as sequence analysis and gene identification in our laboratory [19-21]. The 3' and 5' clone IDs of the EST's are listed in Table 1, columns, 2 and 3 respectively. Those representing a given gene have been grouped in the same or different TC (Table 1, column 4) according to the TIGR database. The different TCs for a given gene annotation possibly represent different family members or perhaps, different regions of the same gene.

The majority of ESTs representing 13 of the genes (*PAL*, *C4H*, *4CL*, *CHS*, *CHR*, *CHI*, *IFS*, *IOMT*, *IFR*, *CCR*, *CAD*, *COMT and CCoAMT*) hybridized complementary RNAs at increasing amounts from 2 to 8 hrs post inoculation in concurrence with the HR as indicated by the ratios marked in bold type. The highest transcript level increases were detected for the ESTs of *CHS, CHR, CHI, IFS1&2, IOMT and IFR1 *genes, all involved in the synthesis of isoflavanone-phytoalexins as shown in Figure 1. Significant but more moderate increases were measured for PAL, C4H, 4CL, CCR, CAD, COMT and CCoAMT ESTs. Enzymes encoded by the corresponding genes function upstream of CHS, and CCR, CAD, COMT and CCoAMT drive the synthesis of lignin. In contrast, transcripts of genes encoding enzymes that function downstream of CHI, directing the synthesis of flavonols, anthocyanins and proanthocyanidins (F3H, DFR, LDOX and FLS) decreased in abundance significantly prior to 8 hrs post inoculation. This is indicated by hybridization ratios lower than 0.67x (1.5 decrease) (in italics) which reflect a decrease in transcript accumulation due to the response to the avirulent pathogen. Interestingly, F3'H transcripts appeared to accumulate significantly by 24 hrs after inoculation according to the microarray data. RNA blots, to be presented later, confirm the observation that F3'H transcripts accumulate much later during the HR response.

The benzoic acid 2-hydroxylase (*BA2H*) gene is predicted to exist in plants such as tobacco, cucumber and potato and encode an enzyme that leads to the synthesis of salicylic acid (SA) from benzoic acid (Figure 1) [22,23]. However, a *BA2H *gene has yet to be identified and cloned from any plant. Consequently, no soybean EST has been annotated as *BA2H *and therefore we could not analyze the overall response of this putative gene during pathogen infection. In *Arabidopsis *it has been shown that SA is synthesized in the chloroplast from chorismate by means of isochorismate synthase (ICS) [24]. Because only one cDNA from this branch of the phenylpropanoid pathway (clone Gm-r1088-2662 annotated as a possible ICS gene) was present on the arrays used in this study and its expression did not significantly change (fdr *p*-value ranged from 0.37 to 0.97) during the course of the experiment, we were unable to analyze categorically the transcript profiles of this possible SA branch of the phenylpropanoid pathway.

Overall, considerable rapid transcript increases occurred for those genes working in the pathway leading to the synthesis of isoflavones/isoflavanones-phytoalexins. These were followed, with lower transcript ratio increments, by those genes involved in lignin biosynthesis. The *F3'H *gene that encodes an enzyme involved in the synthesis of flavones is unique in its response in that it showed a relatively delayed increase in transcript accumulation.

An offshoot of this microarray data analysis was the dual and opposite response for two subsets of ESTs for many of the pathway members, where one cDNA subset showed significant increases in transcript accumulation during the HR while the second subset showed a decrease (see Table 1). In the case of DFR and CCR, the increasing ratios of one of the subsets (bold type) and the decreasing ratios of the second subset (in italics) are so clearly distinct that it suggests the existence of two genes, perhaps two family members with diverse functionality, subcellular localization, or tissue specific activation/regulation. In three instances (CHR, COMT and CCoAMT) ESTs from a specific TC (TC203399, TC203610 and TC225339 respectively) show the reciprocal response setting them apart from the rest of the ESTs with the same annotations.

### Single gene transcript analysis of the phenylpropanoid pathway over a 53-hour time course post inoculation

For a more in depth analysis of the transcriptional activity of the soybean pathogen responsive genes of the phenylpropanoid pathway, RNA blots containing RNAs extracted from inoculated plants at 0, 2, 4, 8, 12, 24, 36, 53 hrs post inoculation were hybridized with single gene EST probes corresponding to cloned, well characterized soybean genes encoding key enzymes of the multiple branches of the phenylpropanoid pathway. An exception to this was the cynnamyl alcohol dehydrogenase (*CAD*) gene that has not been cloned or sequenced in soybean but for which there are multiple soybean ESTs annotated as CAD in the GenBank and TIGR databases. These annotations were based on sequence similarities to *CAD *genes from *Medicago, Arabidopsis*, cowpea, *Oryza sativa *and other species, with an end result of multiple soybean CAD ESTs belonging to multiple tentative contigs (TCs). A soybean EST (AW568106, Gm-r1030-4089, TC 204440) was chosen as a putative representative of a soybean *CAD *gene based on the 88.83% identity and 95.13% similarity of its TC to a *Medicago sativa CAD *ortholog [25].

#### 1. Cynnamylalcohol dehydrogenase (CAD)

Using the putative CAD cDNA clone described above as a probe, the relative transcript abundance of this gene and possibly other CAD family members was determined in portions of leaf tissue harvested at 0, 2, 4, 8, 12, 24, 36 and 53 hrs after inoculating plants with *Psg *with or without *avrB*. To assess the effect of the physical and metabolic stress caused by the infiltration protocol itself on *CAD *induction, a parallel blot with RNAs extracted from leaf tissues infiltrated with MgCl_2 _solution devoid of pathogen was probed and used as the base line reference.

**Figure 2:**
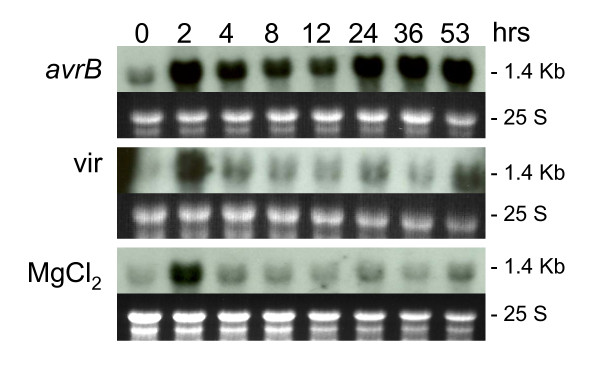
**Cynnamyl alcohol dehydrogenase (CAD) RNA (1.4 kb) profile**. Measurements made at 8 intervals through a 53 hr period in response to infection by *Pseudomonas syringae *pv *glycinea *with *avrB *(*avrB*) or without (vir) (First and second panels respectively). Third panel is the CAD RNA profile in response to infiltration with control medium (MgCl_2_). Dark background sub-panels are the 25 S ribosomal RNAs from corresponding ethidium bromide-stained gels prior to membrane transfer and shown to compare sample loading. Gm-r1030-4089 soybean cDNA clone was used as probe.

Figure 2 summarizes the hybridization results obtained with the *CAD *gene probe. Infiltration with just MgCl_2 _solution induced CAD transcript accumulation by the 2 hr time point decreasing rapidly to almost pre-inoculation (time 0) levels at the 4 hr measurement. In contrast, higher increases in transcript accumulation were observed in tissues infiltrated with *Psg*-*avrB*. The very high transcript accumulation observed at the 2 hr measurement may be in part due to a stress response incited by the vacuum infiltration *per se *since in the MgCl_2 _infiltration control treatment considerable amount of transcript was also measured at 2 hr post infiltration (Figure 2). In *Psg-avrB *infected tissues, the initial expression burst (2 hr) was followed by slight decreases from 4 to 12 hr after which a second increase in transcript accumulation was observed at 24, 36 and 53 hrs. The CAD hybridization to RNAs from tissues infiltrated with the *Ps*g-vir or MgCl_2 _alone, showed similar patterns but with slightly higher intensities with *Psg-vir *RNAs (Figure 2).

The RNA blot hybridization results shown in Figure 2 agree with those obtained in the microarray analysis for a subset of CAD cDNAs showing higher hybridization ratios at 8 and 24 hrs post inoculation relative to values of MgCl_2 _infiltrated plants (Table 1).

#### 2. Chalcone synthase (CHS)

**Figure 3:**
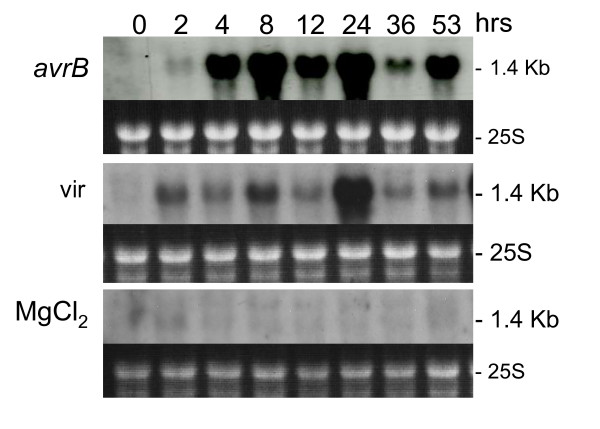
**Chalcone synthase (CHS) RNA (1.4 kb) profile**. Measurements made at 8 intervals through a 53 hr period in response to infection by *Pseudomonas syringae *pv *glycinea *with *avrB *(*avrB*) or without (vir) (First and second panels respectively). Third panel is the CHS RNA profile in response to infiltration with control medium (MgCl_2_). Dark background sub-panels are the 25 S ribosomal RNAs from corresponding ethidium bromide-stained gels prior to membrane transfer and shown to compare sample loading. The soybean CHS genomic clone (pC2H2.0, Gm-b10BB-5) was used as probe.

A genomic CHS cloned fragment (pC2H2.0, Gm-bBB-5 in Table 1), [26] with homology to all 8 *CHS *genes was used in hybridizations to RNA blots containing RNAs extracted from plants infiltrated with 1) avirulent pathogen (*Psg*-*avrB*), 2) virulent pathogen (*Psg*-vir) and 3) MgCl_2 _control solution. As shown in Figure 3, infiltration of leaf tissues with MgCl_2 _alone had very little effect on CHS transcript induction. Plants infiltrated with the virulent pathogen revealed high levels of CHS induction particularly at the 24 hr time measurement. However, plants infiltrated with the avirulent *Psg*-*avrB *displayed much higher levels of hybridization starting as early as 4 hrs post inoculation. These results support the involvement of one or more *CHS *genes in the rapid HR mounted by the plant to defend itself from the invading pathogen, as well as a later involvement of CHS in efforts to defend against a virulent pathogen.

The hybridization results obtained for this genomic (pC2H2.0, Gm-b10BB-5) and other CHS cDNA clones in the microarray experiments (Table 1) are in accordance with the RNA blot data, showing very high hybridization ratios, in some cases, as high as 49 and 64 fold at 8 and 24 hrs post infiltration.

An interesting observation is the lower amounts of CHS transcripts detected at 12 and 36 hrs data points. In those two instances, the leaf tissues from which those RNAs were extracted were harvested at 10:45 and 10:55 PM respectively. Eleven PM was the time at which the lights in the growth chamber turned off. This result indicates that towards the end of the diurnal cycle either there was a reduction in the amount of transcripts being synthesized or that certain CHS transcripts were targeted for degradation.

A delayed induction of *CHS *genes with transcript accumulation that peaked at about 4 hrs after fungal elicitor addition, compared to the more rapid induction of a *CAD *gene showing maximum transcript accumulation at 2 hrs, had been observed also in *Phaseolus vulgaris *cell cultures [27,28]. The faster induction of the *CAD *gene seems to be due to a transient stress response to the vacuum infiltration *per se *as was described earlier (Figure 2). On the other hand, we observed a difference in the sustained high levels of CHS transcript accumulation up to 53 hrs when compared to what had been observed in alfalfa leaves infected with *P. syringae *pv. *pisi*, where the levels of CHS transcripts peaked at 6 hr and declined rapidly to about 20% of peak value by 48 hr post inoculation [29,30].

#### 3. Isoflavone synthase (IFS)

Two soybean *IFS *genes (*IFS1 *and *IFS2*) have been identified, cloned and sequenced [14]. The corresponding EST clones (Gm-c1059-264 and Gm-c1027-870) from the soybean EST collection [31] were chosen to be printed on the soybean cDNA microarrays analyzed in this study and to be used as probes for study with the RNA blots.

**Figure 4:**
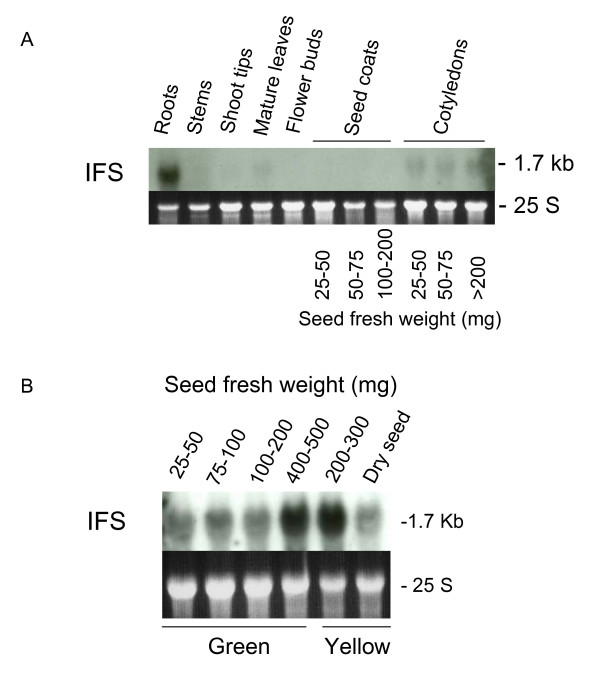
**Isoflavone synthase (IFS) tissue-specific expression in *Glycine max*, cultivar Williams**. (A) IFS transcripts (1.7 kb) detected in a RNA blot containing 10 μg of total RNA samples purified from roots, stems, shoot tips, mature leaves, flower buds, seed coats and cotyledons of soybean plants, cv. Williams 82. Seed coats and cotyledons from three different stages of seed development were used. Seed fresh weight in milligrams is shown at bottom. (B) Expression of IFS in the cotyledon of *Glycine max*, cv. Williams through seed development. The 25 S ribosomal RNAs from the ethidium bromide-stained gel prior to membrane transfer are shown in the dark background sub-panels to compare RNA sample loading. The Gm-c1059-264 soybean cDNA clone was used as probe.

Isoflavone synthase is a key enzyme in the synthesis of soybean isoflavones (daidzein, genistein, glycitein) and the defense inducible phytoalexins such as coumestans and pterocarpans (glyceollins) [32]. *IFS *genes are expressed preferentially in the roots and cotyledons of the soybean plants under normal growing conditions (Figure 4A and 4B). In the cotyledons, the highest level of expression occurs at the time when they transition from the green (fully expanded, 400–500 mg fresh weight) to the yellow (dehydrating, 200–300 mg fresh weight) stages of late cotyledon development during seed maturation (Figure 4B), which precedes the synthesis of isoflavones found when the seeds mature as measured by Dhaubhadel *et al*. [17].

**Figure 5:**
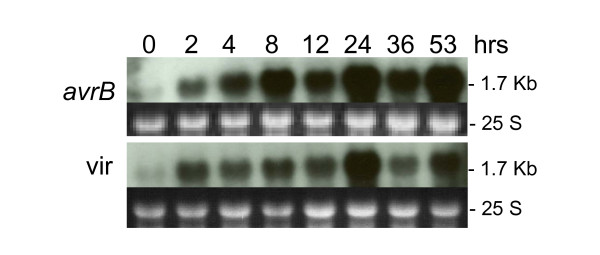
**Isoflavone synthase (IFS) RNA (1.7 kb) profile**. Measurements made at 8 intervals through a 53 hr period in response to infection by *Pseudomonas syringae *pv *glycinea *with *avrB *(*avrB*) or without (vir) (First and second panels respectively). Dark background sub-panels are the 25 S ribosomal RNAs from corresponding ethidium bromide-stained gels prior to membrane transfer and shown to compare sample loading. The Gm-c1059-264 soybean cDNA clone was used as probe.

Figure 5 shows a rapid accumulation of IFS transcripts by 2 hrs after inoculation with *Psg *with or without the *avrB *gene. Although the virulent pathogen incites a response of the *IFS *gene(s), it is not maintained at the same level as the one provoked by the avirulent pathogen. Larger amounts of transcripts were detected after the 4 hr time point in leaves infiltrated with *Psg-avrB *(Figure 5). Similar to what was observed in the CHS transcript accumulation time course, the amount of IFS transcripts was reduced at 12 and 36 hours post inoculation and as mentioned above, the tissues used for the RNA extractions of these two data points were harvested at the end of the diurnal cycle. These results suggest that transcription or transcript accumulation/degradation of *CHS *and *IFS *genes may be affected by the diurnal cycle.

The IFS hybridization results obtained with the RNA blots containing RNAs from plants infected with the avirulent pathogen (Figure 5) are in agreement with those already described in the microarrays experiment section (Table 1). The latter results showed hybridization ratios at 8 and 24 hrs post infiltration much higher than at 2 hrs for those cDNAs representing both *IFS1 *and *IFS2 *genes.

#### 4. Flavonoid 3' hydroxylase (F3'H)

The soybean *F3'H *gene was identified and its expression characterized recently in our laboratory [19]. Two of several complete cDNA clones from the soybean EST collection, Gm-c1012-683 and Gm-c1019-10961, were chosen to represent the *F3'H *gene when printing the microarrays and to be used as probes for the RNA blot analysis of the response of this gene to pathogen infection.

**Figure 6:**
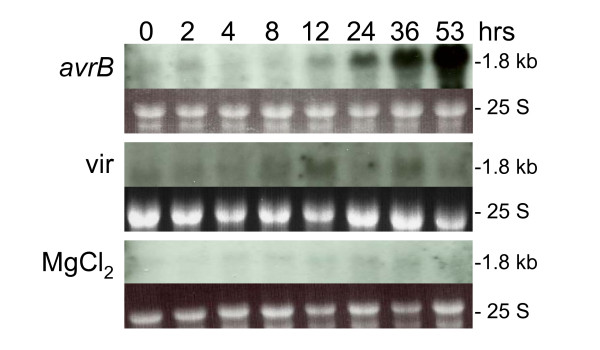
**Flavonoid 3'-hydroxylase (F3'H) RNA (1.8 kb) profile**. Measurements made at 8 intervals through a 53 hr period in response to infection by *Pseudomonas syringae *pv *glycinea *with *avrB *(*avrB*) or without (vir) (First and second panels respectively). Third panel is the F3'H RNA profile in response to infiltration with control medium (MgCl_2_). Dark background sub-panels are the 25 S ribosomal RNAs from corresponding ethidium bromide-stained gels prior to membrane transfer and shown to compare sample loading. Gm-c1019-10961 soybean cDNA clone was used as probe.

The *F3'H *gene was found to be strongly expressed in seed coats at early stages of development and very poorly in all other tissues including the cotyledons [19]. The low level of expression of this gene in the mature leaves can be seen also in the RNA blots shown in Figure 6 at the 0 data points which represent the status of the leaf tissue prior to infiltration with the pathogen or MgCl_2 _alone. Leaves infiltrated with the virulent pathogen showed a slight accumulation of F3'H transcripts compared to those treated with MgCl_2 _alone, but very minimal compared to the larger amounts accumulated by the leaves of plants inoculated with the avirulent pathogen. Starting at about 2–8 hours F3'H accumulated increasingly to high levels at 36–53 hrs post inoculation.

These results suggest that F3'H, an enzyme that adds a hydroxyl group to the 3' position of the B-ring of naringenin and dihydrokaempferol to generate eridictyol and dihydroquercetin respectively, may play a role in the cascade of reactions elicited during defense. Flavones and flavonols are secondary metabolites derived from eridictyol and dihydroquercetin by the intervention of flavone synthases (FS1 and FS2) and flavonol synthase (FLS) respectively (Figure 1). Putative soybean flavanol synthase (FLS) ESTs were printed on the microarrays analyzed in this study but the hybridization ratios 2, 8 and 24 hrs after *Psg-avrB *infection are very low and appeared to decrease with time. This decrease may indicate that flavonols have little or no role in defense. If that were the case in soybean the apparent increase in F3'H transcripts upon inoculation may be directed towards increased synthesis of flavones (apigenin and luteolin). Induction of flavone synthesis has been observed in soybean cell cultures upon osmotic stress, which to some extent mimics the effect of a fungal elicitor [33,34].

#### 5. Flavanone 3 hydroxylase (F3H)

Differential hybridization to soybean microarrays of flower buds RNAs from a pink flower mutant and a purple flower isoline was instrumental in the identification of the *Wp *locus as a flavanone 3 hydroxylase gene [21]. The two cDNA clones that were over expressed in the purple flower, Gm-c1012-683 and Gm-c1019-2646, were used as tools in the identification and characterization of two very similar soybean *F3H *genes (*F3H1 *and *F3H2*). *F3H1 *is expressed strongly in seed coats of *Wp *plants, more weakly in flower buds and shoot tips (meristem and young leaves) but barely detectable expression by RNA blots in cotyledons and roots [21]. In contrast, the *F3H2 *gene does not seem to be expressed, or very weakly, judging by the lack of hybridization to seed coat or flower bud RNAs in the pink flower mutant where a transposon insertion disrupted *F3H1 *[21].

**Figure 7:**
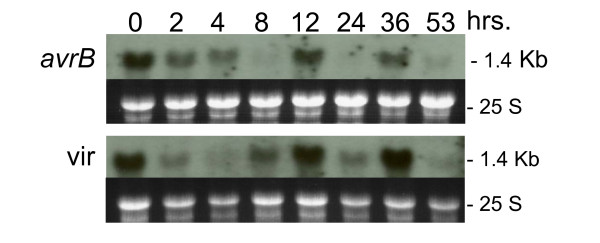
**Flavanone 3-hydroxylase (F3H) RNA (1.4 kb) profile**. Measurements made at 8 intervals through a 53 hr period in response to infection by *Pseudomonas syringae *pv *glycinea *with *avrB *(*avrB*) or without (vir) (First and second panels respectively). Dark background sub-panels are the 25 S ribosomal RNAs from corresponding ethidium bromide-stained gels prior to membrane transfer and shown to compare sample loading. Gm-c1012-683 soybean cDNA clone was used as probe.

**Figure 8:**
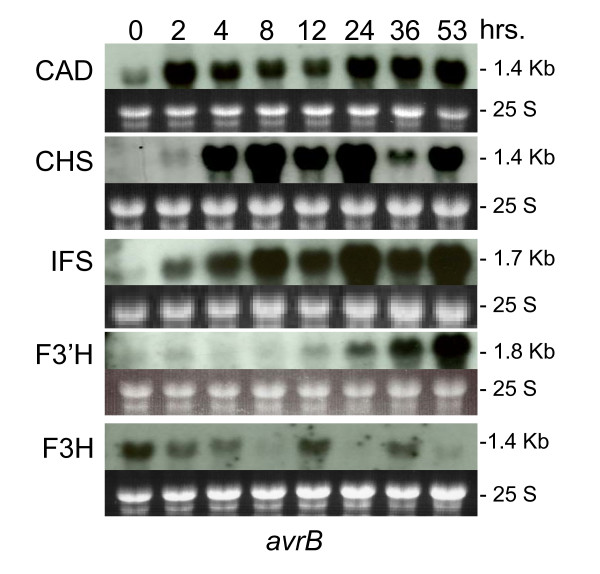
**Alignment of CAD, CHS, IFS, F3'H and F3H, RNA gel blot profiles**. The alignment of the RNA profiles from previous five figures revealed the opposite fluctuation in RNA accumulation of the F3H RNAs during the plant's response to *Pseudomonas syringae *pv. *glycinea *with *avrB *infection.

Using the Gm-c1012-683 cDNA clone as probe, we determined the transcript profile for the *F3H *genes during the 53 hrs period after infiltration with the avirulent or virulent *Psg *(Figure 7). In contrast to the increase in transcript accumulation with time after infection that we had observed for *CAD, CHS, IFS *and *F3'H *genes, the F3H transcripts decreased very rapidly by the 2 hr measurement. More interesting was the fluctuating amounts of these transcripts at different times in the course of the 53 hrs after treatment that is clearly reveled by aligning all 5 RNA blots as shown in Figure 8. The F3H transcripts in the *Psg-avrB *infected tissues decreased for a period of 4 to 8 hrs after infection but at 12 hrs there was an increase. This is also the time at which CHS and IFS transcripts decreased in abundance. At 24 hrs, F3H transcripts decreased again which is the time at which CHS and IFS transcripts were most abundant. This pattern repeats itself at 36 and 53 hrs measurements. Clearly, the regulation of the *F3H *gene expression is different and opposite to that of the *CHS *and *IFS *genes during the defense response.

Further supporting the observation that F3H responds differently than IFS and CHS are the results obtained for the RNAs extracted from tissues infected with the virulent pathogen (Figure 7). At the 8, 12, 24 and 36 hr time points the amount of transcripts hybridizing is higher than in the corresponding RNA samples extracted from plants infected with the avirulent pathogen. A possible explanation for this result is the lesser induction of *IFS *(by the virulent strain) and consequently less IFS competing with F3H (Figure 1).

In addition to the down regulation of the *F3H *gene, the overall abundance of the F3H transcripts was very low. The hybridization intensities from the RNA blot shown in Figure 7 resulted after exposing the autoradiographs for a period of 6 days while the autoradiographs of all other blots (CAD, CHS, IFS and F3'H) were exposed for 3 days.

The hybridization ratios for the F3H cDNAs printed in the microarray also reflect decrease at 8 and 24 hours in HR samples similar to that observed in the RNA blot (Table 1). The two F3H cDNAs were 'control clones' that had been printed 8 times in each array providing 54 data points in the microarray analysis.

To synopsize the results of the RNA blot hybridizations for the 5 gene families (*CAD*, *CHS*, *IFS*, *F3'H *and *F3H*), we have shown that CAD transcripts accumulated at high levels at an earlier time followed by CHS and IFS. With a 12 hr delay F3'H transcripts also accumulated, presenting the first demonstration that F3'H may participate in defense. In contrast, F3H transcripts decreased to the lowest levels at the time when the up-regulated genes accumulated transcripts at their peak values (Figure 8; 8 and 24 hr) suggesting a down regulation or relegation of this gene/enzyme function to those up-regulated HR responders and consequently predicting a lesser role in the resistant response in soybean. Because F3H is an enzyme required for the synthesis of the three classes of anthocyanins and proanthocyanidins and at an early step in their synthetic pathway (Figure 1), this flavonoid class most likely does not play a major role in deterring pathogens in soybean. In support of this statement are the results obtained in the microarray hybridization experiments where two *F3H *downstream genes analyzed, *DFR and LDOX*, also manifested down regulation (Table 1). These results contrast with the observed up-regulation of four putative anthocyanin biosynthesis related ESTs in tomato plants responding to *Psg *infection [8].

### Multiple *CHS *genes are induced in response to pathogen infection

As shown in Figure 3, CHS transcripts were strongly upregulated. Though accumulation of total CHS transcripts in response to pathogen attack has been documented not only by us but many others [16,29,30,35-38], little is known about the gene specific expression profile of the *CHS *family of genes that exist in most legumes, including soybean. Our microarray and RNA blot analyses have demonstrated that there is a significant and rapid increase in CHS transcript accumulation in the incompatible interaction relative to the interaction with the virulent strain, and that *CHS *expression peaks near the 8 hour time point post-infiltration. However, the CHS probes on the cDNA arrays represent long coding regions that will not sufficiently differentiate this highly homologous gene family (>95% sequence identity in the open reading frame for some members).

**Figure 9:**
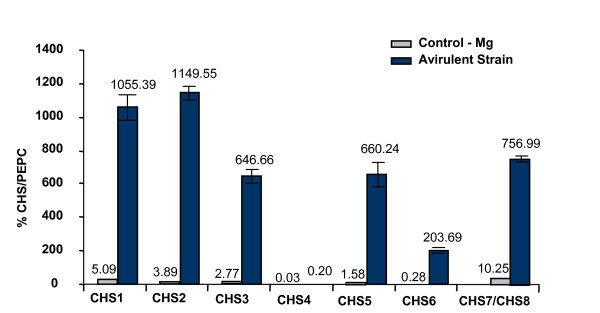
**Expression profile of the *CHS *gene family members as determined by TaqMan^® ^RT-PCR**. Total RNA from leaves of *Glycine max *cv. Williams infiltrated with MgCl_2 _(gray boxes) or the avirulent (black boxes) strains of *Pseudomonas syringae *pv. *glycinea *was isolated after eight hours of infection, reverse transcribed and subjected to real-time PCR. Relative amounts were calculated and normalized with respect to *PEPC *transcript levels (=100%). Data shown represents mean values obtained from three independent amplification reactions and the error bars indicate the S.E. (standard error) of the mean.

In order to identify which *CHS *gene family member(s) responded to the pathogen, we investigated the transcript levels of each individual gene of the eight member gene family using the gene-specific technique of TaqMan RT-PCR. We compared the relative profile of the *CHS *gene family in soybean leaves inoculated with the avirulent, *Psg*-*avrB *strain to those inoculated with MgCl_2 _alone at the 8 hrs post infiltration time point (Figure 9). Our results indicated that the amount of total CHS mRNA was considerably higher in soybean leaves inoculated with the *Psg*-*avrB*, than in those inoculated with the MgCl_2 _alone (Figure 9). These results are consistent with the microarray data in Table 1, the CHS RNA blot (Figure 3) and previous reports on increased total *CHS *expression in soybean leaves infiltrated with an avirulent strain of *Psg *[16]. Interestingly, leaves infected with the avirulent pathogen accumulated higher and diverse levels of all *CHS *gene transcripts except for *CHS4 *(Figure 9). Maximal amounts were detected for *CHS2 *and *CHS1 *in leaves experiencing the incompatible interaction (Figure 9), but expression was greatly increased for all genes except *CHS4*.

## Discussion

Phenylpropanoids (particularly lignin, the isoflavonoid phytoalexins, and flavonoids) serve a variety of structural and metabolic functions and their induction in response to stress has been studied and documented for a few model plant systems [39,40]. *In vivo *labeling has demonstrated marked, but transient increases in rates of synthesis of phytoalexin biosynthetic enzymes concomitant with the onset of phytoalexin accumulation [41-43]. The transient increases in enzyme synthesis reflected increases in the levels of the corresponding mRNA activities [44-46]. In contrast, very little has been concluded regarding gene activities of enzymes of the flavonoid pathway leading to the synthesis of anthocyanins, proanthocyanidins, flavones and flavonols in response to pathogen infection.

Transcript levels of some of these genes have been examined independently one at a time and more recently in global microarray analysis of *Arabidopsis*, tomato, *Medicago truncatula *and soybean plants responding to bacterial and fungal infections. These studies have provided some hints as to which branches of the phenylpropanoid pathway may be activated during the resistance response in those and other plants [7-11]. Based on the up-regulation of a few ESTs reported from those microarray studies it appears that the lignin/suberin branch of the phenylpropanoid pathway is up-regulated in all the plants examined but that branches downstream of CHS (flavonoids and isoflavonoids) appear not to be induced in *Arabidopsis *[12]. In contrast, CHS and anthocyanin pathway ESTs were up-regulated in tomato plants [8] and isoflavonoid ESTs in *Medicago truncatula *and soybean [9-11].

In the present study, a combination of two technologies, cDNA microarrays and RNA gel blot hybridizations, was used to determine the transcript profiles of a set of genes encoding key enzymes involved in the synthesis of secondary metabolites derived from several branches of the soybean phenylpropanoid pathway in response to *Psg *infection. All together, the resulting transcript profiles obtained have shown that lignin/suberin biosyntheis appears to be the first response of the pathway in the infected soybean leaves judging by the higher amounts of CAD transcripts at 2 hrs after *Psg-avrB *inoculation (Figure 8).

Immediately following CAD was the induction of the isoflavonoid branch of the pathway as reflected by higher accumulation of CHS, CHR, CHI, IFS, IOMT, and IFR transcripts 4 hrs post inoculation (Figure 8 and Table 1). The induction of the isoflavonoid pathway was further enhanced during the resistance response elicited by the *Psg-avrB *infection as compared to the induction in response to the virulent strain (Figures 3 and 5 and Table 1). These results support previous observations enlisting *CAD*, *CH*S and *IFS *as early responders [9-12,29,35,37,44,46-48].

A novel finding was the more delayed increase in F3'H transcripts starting at approximately 12 hr after infection with *Psg-avrB *(Figure 8) and clearly distinct from the kinetics of transcript accumulation observed upon infection with the virulent *Psg *(Figure 6). Based on this finding we could speculate that the flavones branch of the flavonoid pathway also participates in the cascade of reactions elicited during defense, although at a later time relative to the isoflavone/phytoalexin branch of the pathway. F3'H hydroxylates naringenin to eriodictyol a substrate for the synthesis of flavones, flavonols, cyaniding anthocyanidins and proanthocyanidins (Figure 1, [21]). Since transcripts of enzymes leading to the synthesis of flavonols, anthocyanidins and proanthocyanidins decreased in abundance in the course of the resistance response, the increase in F3'H transcripts during this same time suggest a potential increase in the synthesis of flavones (i.e. luteolin) and therefore an involvement of these secondary metabolites 8 hrs into the defense response. No soybean or any other plant EST has been annotated as flavone synthase 1 or 2 (FS1 and FS2) and it is not currently possible to determine if transcripts from these genes accumulate in the same fashion as F3'H to further support an involvement of flavones in defense.

In contrast, three other flavonoid branches leading to the synthesis of flavonols, anthocyanins, and proanthocyanidins were slowed down judging by the lower amounts of transcripts hybridizing to the F3H, DFR, LDOX and FLS cDNAs as early as 2 hrs after infection (Figure 8 and Table 1). The suppression of these branches of the flavonoid pathway seems to be lifted somehow in the soybean plant response to infection with the virulent *Psg*. Transcripts of the *F3H *gene encoding the key enzyme feeding all three branches (Figure 1) accumulated slightly higher amounts in plants infected with *Psg *lacking *avr-B *(Figure 7). In tomato, microarray data from a one time point (8 hr) after infection with *P. syringae *pv. *tomato *has shown up-regulation of four putative anthocyanin ESTs but no activation of isoflavonoid ESTs [8].

Even though there have been previous reports describing increases in CHS and IFS transcripts in tissues of infected soybean plants, the cyclic nature of the increments, possibly associated with the diurnal cycle, observed in our RNA gel blot analysis, had not been documented before (Figure 8). Likewise, the increases in F3H transcripts at the times when CSH and IFS transcripts decrease in abundance is a novel finding showing that the synthesis of F3H transcripts is opposite correlated to the activity of the *IFS *genes during the pathogen stress response. This observation allows us to speculate that the biosynthesis of anthocyanins, proanthocyanidins and flavonols is relegated to second place compared to that of the isoflavones/isoflavanones-phytoalexins during the soybean resistance response to *Psg-AvrB *infection. We further infer from the data showing that F3H transcript levels are reciprocally correlated to those of CHS and IFS, that the entrance into the flavonol/anthocyanin/proanthocyanin branch of the pathway is possibly restricted by the low levels of the F3H and transcripts of genes downstream of F3H, thus giving preference to the isoflavone/phytoalexin branch of the pathway during the response to infection. The critical nature of partitioning the naringenin substrate between F3H and IFS enzymes has been noted previously in that F3H must be non-functional in order to permit accumulation of isoflavones in *Arabidopsis *plants transformed with an *IFS *gene [15].

Induction of a key checkpoint enzyme in the pathway, CHS, in response to a pathogen has been reported previously in many plant systems, including soybean [16,29,35-37,31]. However, the relative abundance of the highly homologous eight gene family transcripts of soybean had not been investigated. By using a highly sensitive and gene specific technique, TaqMan RT-PCR, we have demonstrated that all CHS gene family transcripts except one, CHS4, accumulated at varying levels in soybean leaves infiltrated with the avirulent strain of *Psg*.

Stress metabolite production is often correlated with avirulent responses, but is also observed in response to the virulent pathogen. A variety of experiments have shown that genes in the phenylpropanoid pathway, such as *PAL *and *CHS*, are induced to the same extent by virulent and avirulent strains, but that avirulent strains elicit a much more rapid response than virulent strains [11,49-51]. However, the RNA gel blots and cDNA microarray analysis on *CHS *expression in soybean leaves infected with *Psg *presented here (Figure 3) and in alfalfa by Esnault *et. al*. [29], have shown that the avirulent strain is significantly more effective at inducing *CHS *than the virulent strain.

The induction of different *CHS *gene family members in *Glycine ma*x (as in many other legumes) is in striking contrast to the model species, *Arabidopsis*, where the expression of the single copy *CHS *is marginally affected by inoculation with the virulent or avirulent *Psg *strains [52-55]. *Arabidopsis *lacks isoflavonoids phytoalexins [56] and the fact that *CHS *appears not to be consistently induced upon infection is a strong indication that in *Arabidopsis *the flavonoids (anthocyanins, proanthocyanidins, flavones and flavonols) might not play a role in defense. However, there is evidence that a rapid activation of cinnamoyl alcohol dehydrogenase (CAD), the branch point enzyme directed towards lignin biosynthesis, occurs in *Arabidopsis *cultivars infected with *Psg *and *Xanthomonas *[48]. The accumulation of transcripts encoding enzymes in the lignin branch such as CAD seems to be a common rapid response mechanism in all plants examined, including *Arabidopsis*, tomato, *Medicago truncatula *and soybean (Figure 2). Thus, increased *CAD *expression may be a common early plant response to pathogens, presumably leading to increased deposition of lignin, suberin and callose into the cell wall to strengthen barriers against further pathogen ingress.

In many plant species, including soybean, key phenylpropanoid pathway genes like *CHS *are encoded by multiple genes exhibiting greater diversity in their 5' and 3' UTRs than in the protein coding regions. There has been considerable speculation whether this encoding reflects a means for temporal, spatial or stimulus specific regulation of gene expression, or whether it simply allows for increased enzyme production under stress conditions, when expression of the whole gene family is often superimposed upon tissue specific selective expression of a subset of family members [57]. Eight hours after infiltration with the avirulent pathogen, we observed significant accumulation of CHS transcripts encoded by nearly all the gene family members (except for *CHS4*), which potentially correlates to the different isopeptides being translated. Support for this hypothesis comes from earlier work on elicitor-treated soybean cell cultures wherein six CHS isomers have been described [58]. De novo synthesis of up to nine CHS isopeptides has been observed in the elicitor treated bean cell cultures and hypocotyls [59]. Thus it seems more likely that in soybean, expression of the whole *CHS *gene family is superimposed upon tissue specific selective expression following pathogen infection.

## Conclusion

The transcriptional analysis of genes from the multiple branches of the phenylpropanoid pathway has shown that in soybean synthesis of lignin/suberin is one of the early responses to *Pseudomonas syringae *pv. *glycinea *infection as deduced by the high accumulation of CAD transcripts 2 hrs after *Psg-avrB *inoculation. Immediately following the CAD response was the induction of the isoflavonoid biosynthetic pathway judging by the high accumulation of CHS, CHR, CHI, IFS, IOMT, and IFR transcripts 4 hrs post inoculation (Figure 8 and Table 1). The data also allowed us to predict that the synthesis of flavones may participate in the R gene-mediated defensive response, as suggested by the higher levels of F3'H transcripts accumulated 24 hours post infiltration. In contrast, transcripts of genes involved in the synthesis of flavonols, anthocyanins and proanthocyanidins, also pathogen deterring compounds, seem to be down regulated during the periods of time when the genes involved in the synthesis of isoflavonoids are up-regulated. However, it appears that at times when the activity of *IFS *genes decrease, synthesis of flavonols, anthocyanins and proanthocyanins may resume based on the concomitant increases of F3H transcripts which encode an enzyme required for biosynthesis of those secondary metabolites. Lastly, we conclude that the large transcript increases measured for *CHS*, the gene encoding the central enzyme of the phenylpropanoid pathway, is brought about by the additive up-regulation of seven *CHS *family member genes.

## Methods

### Plant material and bacterial inoculation

Soybean [*Glycine max *(L.) Merrill cv Williams 82, RPG1 dominant] plants were grown in an environmentally controlled growth chamber, at constant 22°C and 16 h of light from 7 AM to 11 PM. Plants with emerging first trifoliolate were inoculated approximately 14 days after germination by vacuum infiltration as described [11]. For the inoculum, the bacterial strains *Pseudomonas syringae *pv. *glycinae *Race 4 with or without the avirulence gene *avrB *on plasmid pVB01 were suspended in 10 mM MgCl_2 _at a concentration of 0.02 A_600 _(corresponding to approximately 2 × 10^7 ^colony-forming units ml^-1^). Control plants were infiltrated with a 10 mM MgCl_2 _solution to correct for the effect of vacuum infiltration on gene expression.

### RNA extraction

Unifoliolate-leaves from several infiltrated plants per treatment were harvested at 0, 2, 4, 8, 12, 24, 36 and 53 hours post-inoculation. The leaves were freeze-dried in a Multi-dry lyophilizer (FTS systems) and stored at -20°C. The total RNA samples used in RNA blots were extracted using a phenol-chloroform and lithium chloride precipitation method [60,61]. RNA was stored at -70°C until used.

### Microarrays and data analysis

A detailed description of the three soybean slide sets containing 9,216 cDNA clones each totaling 27,648 used for this study has been presented [18]. Fluorescent labeled cDNA probes were prepared by reverse transcription of total RNA in the presence of amino-allyl-dUTP (Sigma, St. Louis), followed by coupling of either Cy3 or Cy5 dyes (Amersham Pharmacia Biotech, Piscataway, NJ) as previously detailed [11]. A loop design was used and data from two biological replicates were statistically analyzed using R/MAANOVA involving GLOWESS normalization and a linear mixed effects model [11]. Fluorescent intensities values for a subset of the cDNAs from the earlier microarray study [11] were evaluated by averaging the ratios and *p*-values of all spots whose false discovery rate *p*-values were below 0.05 or 0.005 for the 2 hr (T2), 8 hr (T8) and 24 hr (T24) post inoculation, respectively. For genes that were spotted on multiple slides, the spot values were averaged within slide and then across slides.

### RNA gel-blot and DNA probes

RNA (10 μg/sample) was electrophoresed in a 1.2% agarose-3% formaldehyde gel [62]. Size-fractionated RNAs were transferred to Optitran-supported nitrocellulose membrane (Midwest Scientific, Valley Park, MO) by capillary action as described in [62] and cross-linked with UV light (Stratagene, La Jolla, CA). Nitrocellulose RNA blots were prehybridized, hybridized, washed and exposed to Hyperfilm (Amersham, Arlington Heights, IL) as described by [63]. All the RNA blot results presented are from autoradiographs exposed for 3 days except for those of blots hybridized with F3H gene probes that were exposed for 6 days.

Cloned DNAs used as probes were digested from their vectors or PCR amplified, electrophoresed, and purified from agarose using the QIAquick gel extraction kit (QIAGEN, Valencia, CA). DNA concentration of the final eluate was determined with a NanoDrop (NanoDrop Technologies, Inc. Rockland, DE). Purified DNA fragments (25–250 ng) were labeled with [α-^32^P]dATP by random primer reaction [64].

### Real Time Quantitative RT-PCR

The highly sensitive TaqMan RT-PCR assay was used to measure the relative levels of the *CHS *gene family members with gene specific probes. Briefly, total RNA was purified using the DNA-free kit (Ambion, Austin, TX) and reverse transcribed using the Superscript II first strand cDNA system for RT-PCR (Invitrogen, Carlsbad, CA) according to the manufacturer's instructions. The resulting cDNA samples were pooled and stored at -20°C.

TaqMan^® ^RT-PCR assays for the target gene were performed in triplicate on cDNA samples or no RT control samples on an ABI Prism 7700 Sequence Detection System (Applied Biosystems, Foster City, CA). Gene specific CHS primers and probes were used as listed in [20]. Parallel amplifications using the same cDNA pools were carried out using primers and probes to the endogenous reference and normalizer, *PEPC16*, which had shown to be expressed at similar levels in leaves inoculated with the two different strains in our earlier optimization experiments (data not presented). From the pooled cDNA, 2 μl of the RT reaction was used as a template in a 25 μl PCR reaction containing 1x TaqMan buffer, 0.4 μM forward and reverse primers, 0.2 μM probe, 0.2 mM dATP, dCTP and dGTP, 0.4 mM dUTP and 3.5 mM MgCl_2 _and 0.025 U/μl AmpliTaq Gold DNA polymerase. The PCR thermal cycling parameters were 50°C for 2 min., 95°C for 10 min followed by 40 cycles of 95°C for 15 sec., 60°C for 1 min. This experiment was replicated thrice. Data were captured as amplification plots. Transcript levels of the *CHS *gene family members were measured relative to the endogenous reference *PEPC*. All calculations and statistical analysis were performed as described in the ABI 7700 Sequence Detection System User Bulletin #2 (Applied Biosystems, Foster City, CA).

## Authors' contributions

GZ identified ESTs of soybean phenylpropanoid genes printed as the 'control clones' in the soybean cDNA microarray chips, created list of all ESTs in the soybean arrays annotated as phenylpropanoid pathway genes, performed the RNA blots, executed the critical analysis of the combined microarray and RNA blots results and wrote the manuscript. JZ performed the microarray hybridizations that generated the microarray data analyzed in this study. JT selected ESTs to represent all CHS family members in the arrays and performed, analyzed and described the TaqMan RT-PCR experiment which determined the CHS family member genes responding to the pathogen. DOG contributed to the design, construction, and production of the soybean cDNA microarray chips. SC directed the microarray hybridization experiments that generated the data analyzed in this study, summarized the microarray data to assemble Table 1, and provided the infected tissues used to extract RNA for the Northern blots and TaqMan experiment. LV directed the development and construction of the cDNA soybean microarrays, provided overall project coordination, and edited the manuscript. All authors critically reviewed the manuscript.

## Figures and Tables

**Table 1:** Soybean phenylpropanoid pathway genes responsive to infection by Pseudomonas syringae with (HR) or without (VIR) avrB

				**Ratio treatment vs MgCl_2 _control^a^**					
				
**Gene Description**	**3'-Clone ID**	**5'-Clone ID**	**5' or 3' TIGR TC**	**T2HR**	**T8HR**	**T24HR**	**T2VIR**	**T8VIR**	**T24VIR**
Phenylalanine ammonia-lyase (PAL)	Gm-r1088-2170	Gm-c1027-2831	TC225162		**2.46**	**1.93**			
Phenylalanine ammonia-lyase (PAL)	Gm-r1070-3081	Gm-c1015-4781	TC216854		**6.23**	**3.81**			**2.07**
Phenylalanine ammonia-lyase (PAL)	Gm-r1021-3996	Gm-c1004-9203	TC225162		**2.44**	**2.26**	1.05	**1.68**	
Phenylalanine ammonia-lyase (PAL)	Gm-b10BB-2	Gm-c1062-1285	TC225162		**3.39**	**2.80**	**2.67**	**2.19**	
Phenylalanine ammonia-lyase (PAL)	Gm-r1083-3853	Gm-c1028-6208	TC225168		**3.39**	**2.62**			
Phenylalanine ammonia-lyase (PAL)	Gm-r1070-4771	Gm-c1016-5168	NA		**3.18**	**3.14**			
									
Cinnamate 4 hydroxylase (C4H)	Gm-r1021-162	Gm-c1004-446	TC204339		**4.76**	**1.67**		**2.25**	
Cinnamate 4 hydroxylase (C4H)	Gm-r1021-1649	Gm-c1004-3560	TC204341		**1.97**				
Cinnamate 4 hydroxylase (C4H)	Gm-r1021-3590	Gm-c1004-8721	TC204341		**2.58**	**1.59**	1.25	**1.89**	
Cinnamate 4 hydroxylase (C4H)	Gm-r1083-1942	Gm-c1028-893	TC204341		**3.43**	**1.93**			
									
4-coumarate:CoA ligase (4CL)	Gm-r1021-2611	Gm-c1004-5129	TC205641		**2.13**				
4-coumarate:CoA ligase (4CL)	Gm-r1083-3061	Gm-c1028-3891	TC205641		**3.20**			**1.84**	
4-coumarate:CoA ligase (4CL)	Gm-r1083-3414	Gm-c1028-4979	TC205641		**2.01**		**1.97**		
4-coumarate:CoA ligase (4CL)	Gm-r1070-5094	Gm-c1016-6727	TC216082		**1.67**	**2.50**			**2.17**
4-coumarate:CoA ligase (4CL)	Gm-r1021-2223	Gm-c1004-4431	TC216190		**1.75**	**1.49**			
4-coumarate:CoA ligase (4CL)	Gm-r1021-3419	Gm-c1004-7053	TC216190		**2.36**	**1.61**		**1.65**	
4-coumarate:CoA ligase (4CL)	Gm-r1070-3141	Gm-c1015-4689	TC216191		**‡**	**9.45**		**1.80**	
4-coumarate:CoA ligase (4CL)	Gm-r1070-7534	Gm-c1029-1150	TC220028		**3.23**	**2.89**		**1.54**	**1.88**
4-coumarate:CoA ligase (4CL)	Gm-r1083-4331	Gm-c1028-7642	TC224198		**2.93**	**1.57**			
4-coumarate:CoA ligase (4CL)	Gm-r1070-3331	Gm-c1015-4673	TC225974		**6.32**			**2.53**	
4-coumarate:CoA ligase (4CL)	Gm-r1083-2896	Gm-c1028-3421	TC226636		**2.60**	**2.20**			
4-coumarate:CoA ligase (4CL)	Gm-r1083-4078	Gm-c1028-6958	TC226636		**5.28**	**2.13**	1.28	**2.16**	
4-coumarate:CoA ligase (4CL)	Gm-r1070-2770	Gm-c1015-4573	TC226637		**7.06**	**4.96**		**1.56**	
4-coumarate:CoA ligase (4CL)	Gm-r1070-2942	Gm-c1015-3925	NA		**1.77**	**2.36**		**1.53**	**1.85**
4-coumarate:CoA ligase (4CL)	Gm-r1083-1803	Gm-c1013-3118	TC229241		*0.13*	*0.43*			
4-coumarate:CoA ligase (4CL)	Gm-r1083-779	Gm-c1009-3084	TC221808		*0.34*	*0.36*			
4-coumarate:CoA ligase (4CL)	Gm-r1070-515	Gm-c1008-190	TC223132		*0.37*	*0.29*		*0.61*	*0.54*
									
Chalcone synthase (CHS)	*Gm-b10BB-5	Genomic clone	NA		**36.35**	**13.22**	**1.83**	**12.47**	**7.15**
Chalcone synthase (CHS)	Gm-b10BB-6	Gm-c1004-1721	TC203259	1.07	**48.80**	**63.70**	**3.31**	**20.48**	**16.68**
Chalcone synthase (CHS)	Gm-r1021-84	Gm-c1004-874	TC203259		**30.70**	**37.53**	**1.49**	**9.06**	**7.21**
Chalcone synthase (CHS)	Gm-b10BB-7	Gm-c1004-2866	TC203319	**4.92**	**34.39**	**50.20**	**2.07**	**14.32**	**15.41**
Chalcone synthase (CHS)	Gm-r1083-83	Gm-c1009-670	TC209602		**48.84**	**33.82**	**1.61**	**10.48**	**11.39**
Chalcone synthase (CHS)	Gm-r1088-5931	Gm-c1065-3606	TC209886		**20.25**	**15.56**		**3.25**	
Chalcone synthase (CHS)	Gm-b10BB-4	Gm-c1028-2741	TC214314		**60.58**	**23.88**	**1.84**	**17.92**	**9.59**
Chalcone synthase (CHS)	Gm-r1070-3019	Gm-c1015-4593	TC214387	2.16	**36.76**	**17.75**	**1.87**	**6.77**	**2.91**
Chalcone synthase (CHS)	Gm-b10BB-3	Gm-c1013-1524	TC214443		**44.57**	**17.35**	**1.60**	**10.28**	**6.15**
Chalcone synthase (CHS)	Gm-r1083-556	Gm-c1009-2808	TC226663		**23.10**	**20.97**	**1.45**	**7.36**	**8.40**
Chalcone synthase (CHS)	Gm-r1083-853	Gm-c1009-4010	NA		**30.91**	**31.56**		**5.24**	**4.41**
									
Chalcone reductase (CHR)	Gm-r1021-1257	Gm-c1004-2376	TC225256		**11.39**	**31.12**		**3.36**	**3.48**
Chalcone reductase (CHR)	Gm-r1021-720	Gm-c1004-1818	TC215169		**7.01**	**18.77**		**3.94**	**2.20**
Chalcone reductase (CHR)	Gm-r1021-4150	Gm-c1004-8921	TC215169		**12.17**	**23.51**		**3.93**	**4.24**
Chalcone reductase (CHR)	Gm-r1021-2517	Gm-c1004-5030	TC215169		**8.94**	**18.25**		**4.82**	**3.20**
Chalcone reductase (CHR)	Gm-r1021-754	Gm-c1004-1771	TC225256		**8.82**	**32.00**		**3.14**	**2.38**
Chalcone reductase (CHR)	Gm-r1088-2718	Gm-c1027-5959	TC203399		*0.23*	*0.13*			
Chalcone reductase (CHR)	Gm-b10BB-36	Gm-c1062-753	TC203399		*0.11*	*0.10*		*0.61*	*0.41*
									
Chalcone isomerase (CHI)	Gm-r1021-1566	Gm-c1004-3954	TC205496		**21.11**	**10.70**	**1.71**	**5.82**	**2.03**
Chalcone isomerase (CHI)	Gm-r1021-154	Gm-c1004-613	TC205496		**10.93**	**6.28**	**1.41**	**3.68**	
Chalcone isomerase (CHI)	Gm-r1083-3171	Gm-c1028-4494	TC205496		**6.87**	**3.20**		**2.00**	
Chalcone isomerase (CHI)	Gm-r1021-1440	Gm-c1004-3529	TC205536		**8.63**	**13.45**		**4.63**	**3.58**
Chalcone isomerase (CHI)	Gm-b10BB-9	Gm-c1026-2606	TC205536		**11.42**	**12.79**	**9.96**	**6.30**	**7.47**
Chalcone isomerase (CHI)	Gm-r1083-43	Gm-c1009-620	TC206681		**12.30**	**16.45**		**5.28**	**4.32**
Chalcone isomerase (CHI)	Gm-r1070-1290	Gm-c1010-653	TC216488		**2.89**	**3.94**			**1.85**
Chalcone isomerase (CHI)	Gm-r1083-4382	Gm-c1028-7808	TC216488		**20.68**	**7.21**		**2.51**	**3.23**
Chalcone isomerase (CHI)	Gm-r1088-8117	Gm-c1072-871	TC227583		**5.17**	**4.23**		**2.06**	
Chalcone isomerase (CHI)	Gm-r1070-3127	Gm-c1015-4783	NA		**2.14**	**2.39**			
									
Isoflavone synthase (IFS1)	*Gm-10BB-30	Gm-c1059-264	NA		**11.09**	**8.35**	**1.39**	**5.40**	**3.79**
Isoflavone synthase (IFS1)	Gm-r1088-8309	Gm-c1072-1106	TC204648		**4.72**			**2.14**	
Isoflavone synthase (IFS1)	Gm-r1021-1181	Gm-c1004-3075	TC215321		**7.01**	**5.82**	**1.47**	**5.03**	**3.23**
Isoflavone synthase (IFS1)	Gm-r1088-1867	Gm-c1027-2050	TC218468		**3.94**	**4.50**		**2.25**	
									
Isoflavone synthase (IFS2)	*Gm-b10BB-29	Gm-c1027-870	TC204612		**17.30**	**13.84**	**1.61**	**8.12**	**7.06**
Isoflavone synthase (IFS2)	Gm-r1083-3218	Gm-c1028-4877	TC204612		**15.24**	**20.97**	**1.37**	**6.41**	**6.82**
									
isoflavone 7-o-methyltransferase (IOMT)	Gm-r1021-2837	Gm-c1004-6344	TC206764		**53.45**	**48.17**		**5.35**	**7.16**
isoflavone 7-o-methyltransferase (IOMT)	Gm-r1088-8483	Gm-c1074-35	TC206765		**58.89**	**112.99**		**8.28**	**19.29**
isoflavone 7-o-methyltransferase (IOMT)	Gm-r1088-8769	Gm-c1074-599	TC210019		**78.25**	**95.67**		**4.14**	
isoflavone 7-o-methyltransferase (IOMT)	Gm-r1021-435	Gm-c1004-1004	TC215839		**5.28**	**4.89**	**1.40**	**4.23**	**4.06**
isoflavone 7-o-methyltransferase (IOMT)	Gm-r1021-2886	Gm-c1004-6334	TC215839		**9.06**	**10.27**	**1.84**	**4.50**	**7.89**
									
Isoflavone reductase (IFR1)	Gm-r1021-2052	Gm-c1004-4918	TC206041		**15.45**	**9.58**	**1.47**	**6.11**	**5.13**
Isoflavone reductase (IFR1)	Gm-r1070-7511	Gm-c1023-366	TC207832		**16.00**	**4.00**			
Isoflavone reductase (IFR1)	Gm-b10BB-33	Gm-c1019-3319	TC207832		**24.67**	**31.57**	**2.81**	**67.71**	**49.21**
Isoflavone reductase (IFR1)	Gm-r1088-4139	Gm-c1027-9301	TC215002		**14.42**	**24.76**			
Isoflavone reductase (IFR1)	Gm-r1088-8260	Gm-c1072-1589	TC215002		**15.35**	**7.52**			
Isoflavone reductase (IFR1)	Gm-r1070-6760	Gm-c1019-3354	TC215005		**50.21**	**55.33**		**2.89**	**18.64**
Isoflavone reductase (IFR1)	Gm-r1021-3760	Gm-c1004-8266	TC226270		**41.64**	**43.11**		**6.50**	**6.77**
Isoflavone reductase (IFR1)	Gm-r1083-4951	Gm-c1028-8510	TC226270		**28.84**	**32.22**		**5.90**	**4.56**
									
Flavonoid 3'-hydroxylase (F3'H)	Gm-b10BB-22	Gm-c1053-348	TC216289		1.47	**1.96**	‡	0.80	
Flavonoid 3'-hydroxylase (F3'H)	*Gm-b10BB-23	Gm-c1019-10961	NA		*0.45*	**1.70**	0.76		
									
Flavanone 3-hydroxylase (F3H)	*Gm-b10BB-12	Gm-c1012-683	TC226020		*0.06*	*0.07*			
Flavanone 3-hydroxylase (F3H)	Gm-b10BB-13	Gm-c1019-2646	TC226020		*0.24*	*0.18*			0.67
									
Dihydroflavonol 4-reductase (DFR)	Gm-r1070-8296	Gm-r1030-1389	TC225293		*0.17*	*0.26*			
Dihydroflavonol 4-reductase (DFR)	Gm-r1021-3500	Gm-c1004-8647	TC225293		*0.17*	*0.26*			
Dihydroflavonol 4-reductase (DFR)	Gm-r1083-1327	Gm-c1013-1350	TC225294		*0.16*	*0.14*			*0.50*
Dihydroflavonol 4-reductase (DFR)	Gm-r1088-7258	Gm-c1067-1717	TC234668		*0.12*	*0.20*			
Dihydroflavonol 4-reductase (DFR)	Gm-r1070-7401	Gm-c1019-4941	TC210161		**4.63**	**9.99**			**5.50**
Dihydroflavonol 4-reductase (DFR)	Gm-r1083-2198	Gm-c1028-1633	TC218842		**4.92**				
									
Leucoanthocyanidin dioxygenase (LDOX)	Gm-r1070-1246	Gm-c1010-216	TC217747						
Leucoanthocyanidin dioxygenase (LDOX)	Gm-r1070-7484	Gm-c1029-932	TC214271		*0.38*	*0.35*			
Leucoanthocyanidin dioxygenase (LDOX)	Gm-r1021-2183	Gm-c1004-4454	TC232276		1.39			1.48	
									
Flavonol synthase (FLS)	Gm-b10BB-15	Gm-c1016-13124	NA		*0.51*	*0.52*			
Flavonol synthase (FLS)	Gm-r1070-4167	Gm-c1016-3181	TC208467			*0.51*			
Flavonol synthase (FLS)	Gm-r1083-823	Gm-c1009-3205	TC218780		*0.21*				
									
Cynnamoyl CoA reductase (CCR)	Gm-r1021-2461	Gm-c1004-5052	TC226044		**4.00**	**2.66**		**2.71**	**1.56**
Cynnamoyl CoA reductase (CCR)	Gm-r1021-523	Gm-c1004-1463	TC204998		**3.63**				
Cynnamoyl CoA reductase (CCR)	Gm-r1021-2593	Gm-c1004-5056	TC226045		**3.39**	**2.64**		**3.34**	**1.79**
Cynnamoyl CoA reductase (CCR)	Gm-r1070-3838	Gm-c1016-1700	TC226917		**7.41**			**2.14**	
Cynnamoyl CoA reductase (CCR)	Gm-r1088-8005	Gm-c1072-6	TC233163		**5.82**	**3.27**			
Cynnamoyl CoA reductase (CCR)	Gm-r1070-1367	Gm-c1008-2980	TC207664		*0.10*	*0.10*			*0.61*
Cynnamoyl CoA reductase (CCR)	Gm-r1070-914	Gm-c1008-1235	TC225292		*0.16*	*0.15*			*0.44*
Cynnamoyl CoA reductase (CCR)	Gm-r1070-2653	Gm-c1015-2660	TC225293		*0.19*	*0.15*			*0.43*
									
Cynnamyl alcohol dehydrogenase (CAD)	Gm-r1083-4523	Gm-c1028-7532	TC225589		**2.13**	**1.99**		**8.06**	
Cynnamyl alcohol dehydrogenase (CAD)	Gm-r1021-3795	Gm-c1004-7796	TC204437		**1.51**	**1.93**	**1.27**	**1.34**	**1.32**
Cynnamyl alcohol dehydrogenase (CAD)	Gm-r1083-2221	Gm-c1028-695	TC206457		**12.73**	*0.56*		**2.07**	**3.36**
Cynnamyl alcohol dehydrogenase (CAD)	Gm-r1021-1331	Gm-c1004-3272	TC225589			**1.52**			
Cynnamyl alcohol dehydrogenase (CAD)	Gm-r1021-2196	Gm-c1004-5210	TC225589		**2.25**	**2.55**			
Cynnamyl alcohol dehydrogenase (CAD)	Gm-r1070-5855	Gm-c1019-927	TC227395		0.73	0.72	0.73		*0.56*
Cynnamyl alcohol dehydrogenase (CAD)	Gm-r1083-1322	Gm-c1013-1592	TC230620		0.82	*0.56*			*0.60*
Cynnamyl alcohol dehydrogenase (CAD)	Gm-r1083-3802	Gm-c1028-6101	TC208568		*0.39*	*0.32*		*0.56*	*0.59*
Cynnamyl alcohol dehydrogenase (CAD)	Gm-r1083-4013	Gm-c1028-6963	TC225589		*0.24*	*0.30*			
									
Caffeic acid O methyltransferase (COMT)	Gm-r1083-548	Gm-c1009-2413	TC212974			**2.25**		0.76	
Caffeic acid O methyltransferase (COMT)	Gm-r1083-3458	Gm-c1028-5510	TC219214		**1.54**	**1.89**		1.46	**1.52**
Caffeic acid O methyltransferase (COMT)	Gm-r1021-973	Gm-c1004-2186	TC226262			**2.01**			
Caffeic acid O methyltransferase (COMT)	Gm-r1083-2739	Gm-c1028-2911	TC226263			**2.07**		**1.52**	1.28
Caffeic acid O methyltransferase (COMT)	Gm-r1083-3457	Gm-c1028-5374	TC226265		**2.30**	**2.85**		**1.72**	**1.65**
Caffeic acid O methyltransferase (COMT)	Gm-r1083-2949	Gm-c1028-2922	TC226997		**11.31**	**9.85**			
Caffeic acid O methyltransferase (COMT)	Gm-r1070-1306	Gm-c1008-2760	TC203610		*0.19*	*0.09*			
Caffeic acid O methyltransferase (COMT)	Gm-r1070-1869	Gm-c1015-70	TC203610		*0.18*	*0.10*			
Caffeic acid O methyltransferase (COMT)	Gm-r1070-3522	Gm-c1016-846	TC203610		*0.14*	*0.10*			
									
Caffeoyl Co-A transferase (CCoAMT)	Gm-r1021-3573	Gm-c1004-7957	TC204613		**1.85**			**1.88**	**1.54**
Caffeoyl Co-A transferase (CCoAMT)	Gm-r1070-3714	Gm-c1016-1000	TC204614		**1.92**			**1.96**	
Caffeoyl Co-A transferase (CCoAMT)	Gm-r1070-6767	Gm-c1019-3293	TC213310		**4.79**				
Caffeoyl Co-A transferase (CCoAMT)	Gm-r1083-141	Gm-c1009-796	TC215141		**3.46**	**2.06**	**1.14**	**2.73**	**2.62**
Caffeoyl Co-A transferase (CCoAMT)	Gm-r1083-4593	Gm-c1028-8263	TC225342		**1.93**				
Caffeoyl Co-A transferase (CCoAMT)	Gm-r1021-1921	Gm-c1004-4301	TC225343		**1.67**	**2.41**			
Caffeoyl Co-A transferase (CCoAMT)	Gm-r1070-6093	Gm-c1019-1156	TC228561		**2.69**	**6.87**			**3.48**
Caffeoyl Co-A transferase (CCoAMT)	Gm-r1070-5202	Gm-c1016-6589	TC228561		**2.13**	**2.17**			
Caffeoyl Co-A transferase (CCoAMT)	Gm-r1088-8678	Gm-c1074-1097	TC228993		**4.69**	**18.13**			
Caffeoyl Co-A transferase (CCoAMT)	Gm-r1083-956	Gm-c1009-4174	TC225339		*0.62*	*0.50*			
Caffeoyl Co-A transferase (CCoAMT)	Gm-r1021-427	Gm-c1004-1316	TC225339		*0.45*	*0.44*			0.83
